# Repurposing Doxycycline to Overcome High‐Glucose–Induced Mitochondrial Biogenesis–Mediated Chemoresistance in Colorectal Cancer Cells

**DOI:** 10.1111/jcmm.70795

**Published:** 2025-10-13

**Authors:** Chang‐Han Wu, Ching‐Wen Huang, Yen‐Cheng Chen, Kwan‐Ling Yip, Zhi‐Feng Miao, Wei‐Chih Su, Tsung‐Kun Chang, Hsiang‐Lin Tsai, Yung‐Sung Yeh, Hsiao‐Sheng Liu, Jaw‐Yuan Wang

**Affiliations:** ^1^ Division of Colorectal Surgery, Department of Surgery Kaohsiung Medical University Hospital, Kaohsiung Medical University Kaohsiung Taiwan; ^2^ Department of Surgery, Faculty of Medicine, College of Medicine Kaohsiung Medical University Kaohsiung Taiwan; ^3^ Graduate Institute of Clinical Medicine, College of Medicine Kaohsiung Medical University Kaohsiung Taiwan; ^4^ Department of Surgery, Faculty of Post‐Baccalaureate Medicine, College of Medicine Kaohsiung Medical University Kaohsiung Taiwan; ^5^ Division of Colorectal Surgery, Department of Surgery Kaohsiung Medical University Ganhshan Hospital, Kaohsiung Medical University Kaohsiung Taiwan; ^6^ Division of Trauma and Surgical Critical Care, Department of Surgery Kaohsiung Medical University Hospital, Kaohsiung Medical University Kaohsiung Taiwan; ^7^ Department of Emergency Medicine, Faculty of Post‐Baccalaureate Medicine, College of Medicine Kaohsiung Medical University Kaohsiung Taiwan; ^8^ Graduate Institute of Injury Prevention and Control, College of Public Health Taipei Medical University Taipei Taiwan; ^9^ Master of Science Program in Tropical Medicine, College of Medicine Kaohsiung Medical University Kaohsiung Taiwan; ^10^ Medical Research Center, Kaohsiung Medical University Hospital Kaohsiung Medical University Kaohsiung Taiwan; ^11^ Center for Cancer Research Kaohsiung Medical University Kaohsiung Taiwan; ^12^ Graduate Institute of Medicine, College of Medicine Kaohsiung Medical University Kaohsiung Taiwan

**Keywords:** chemoresistance, doxycycline repurpose, hyperglycaemia, mitochondrial biogenesis, p‐PGC‐1α overexpression

## Abstract

Chemoresistance is a major contributor to treatment failure in most patients with cancer. Hyperglycaemia enhances chemoresistance in stage III colorectal cancer (CRC) patients, potentially through a mechanism involving c‐Myc. Phospho‐PGC‐1α (p‐PGC‐1α), a transcription coactivator, regulates energy metabolism with c‐Myc and is a key regulator of mitochondrial biogenesis. We hypothesised that high glucose (HG) promotes mitochondrial biogenesis by upregulating c‐Myc and p‐PGC‐1α, thus enhancing chemoresistance in CRC cells, and that inhibiting mitochondrial biogenesis alleviates this chemoresistance. In vitro, HG significantly increased mitochondrial mass (*p* < 0.001), oxygen consumption rate (*p* < 0.001), cell migration (*p* < 0.05) and oxaliplatin resistance (*p* < 0.001) in LoVo and HCT116 cells. p‐PGC‐1α and COX4 protein expression were increased in the HG and oxaliplatin‐resistance groups in LoVo and HCT116 cells (all *p* < 0.001) and decreased in the doxycycline group (*p <* 0.001). In vivo, doxycycline combined with oxaliplatin more notably reduced tumour volume than oxaliplatin alone in hyperglycaemic BALB/c nude mice (*p* < 0.05). c‐Myc, p‐PGC‐1α and COX4 protein expression were significantly higher in tissues with CRC and hyperglycaemia who experienced relapse than in those with CRC and normoglycaemia who did not experience relapse (all *p* < 0.05). Overall, this study demonstrated that HG upregulates p‐PGC‐1α and COX4 expression to enhance oxaliplatin resistance by promoting mitochondrial biogenesis and indicates that doxycycline can overcome the chemoresistance induced by HG. Repurposing of doxycycline might reduce chemoresistance in hyperglycaemic CRC patients receiving adjuvant chemotherapy.

## 1 Introduction

Chemoresistance is a major factor contributing to treatment failure in most patients with cancer, including those with colorectal cancer (CRC). Despite surgical resection and adjuvant chemotherapy being standard treatments, some patients receiving oxaliplatin‐based adjuvant therapy ultimately experience recurrence or metastasis, which presents a major challenge in the effective treatment of CRC [1, 2, 3, 4]. Moreover, hyperglycaemia may adversely impact clinical outcomes in stage III CRC patients undergoing adjuvant chemotherapy. The mechanism of oxaliplatin resistance could involve enhanced phosphorylation of two glycolysis‐related target proteins, SMAD3 and c‐Myc, along with upregulation of EHMT2 expression [5]. Metformin (1,1‐dimethylbiguanide hydrochloride), an oral hypoglycaemic agent widely prescribed for type 2 diabetes, has also been suggested as an adjunct to enhance the chemosensitivity of CRC cells to 5‐fluorouracil and oxaliplatin both in vitro and in vivo [6]. High glucose (HG) may exacerbate CRC chemoresistance, but modulating the underlying mechanisms could reduce HG‐induced chemoresistance.

Mitochondrial biogenesis is a crucial cellular process in which damaged mitochondria are renewed; it supports the biosynthetic and bioenergetic needs of a cell and plays a vital role in tumorigenesis [7]. Mitochondrial biogenesis refers to the increase in mitochondrial mass from pre‐existing mitochondria, regulated by the co‐transcriptional factor peroxisome proliferator‐activated receptor gamma coactivator‐1α (PGC‐1α) and other associated factors [8]. c‐Myc aberrations or the upregulation of c‐Myc‐related pathways through alternative mechanisms occur in the majority of cancers [9]. The oncogenic transcription factor c‐Myc is overexpressed in many types of cancer and is a typical oncogene on which many cancers are highly dependent. c‐Myc is essential for the rapid proliferation of cancer cells [10] and promotes chemoresistance and tumour growth in CRC [11]. c‐Myc regulates genes associated with ribosome and mitochondrial biogenesis, as well as glucose and glutamine metabolism [12]. Sancho et al. proposed a c‐Myc/PGC‐1α balance model to explain the high energy metabolism in cancer stem cells [13]. PGC‐1α is a transcriptional coactivator that is highly expressed in mitochondria and tissues with high energy demands and essential roles in energy metabolism, mitochondrial biogenesis, homeostasis and various biological pathways [14, 15, 16, 17].

Recent studies further emphasise the pivotal role of mitochondrial function in cancer biology and therapy. Li et al. summarised the anticancer potential of tetracycline antibiotics, highlighting their ability to impair mitochondrial biogenesis and interfere with cancer cell proliferation [18]. Additionally, Delgado‐Navarro et al. demonstrated that doxycycline effectively inhibited prostate cancer progression by impairing mitochondrial respiration and reducing tumour proliferation in preclinical models [19]. These findings support the therapeutic rationale for repurposing mitochondrial inhibitors in oncology.

On the basis of the aforementioned assumption, we hypothesise that hyperglycaemia enhances chemoresistance in stage III CRC patients through a mechanism involving c‐Myc expression. PGC‐1α regulates energy metabolism in coordination with c‐Myc and is described as an important regulator of mitochondrial biogenesis. In the present study, we investigated whether hyperglycaemia increases the chemoresistance of CRC cells and whether this chemoresistance can be reduced by combining doxycycline with chemotherapeutic agents. In particular, we examined whether doxycycline enhances the therapeutic efficacy of oxaliplatin in CRC cells both in vitro and in vivo, and elucidated the mechanism through which the repurposing of doxycycline through inhibition of mitochondrial biogenesis can overcome chemoresistance.

## 2 Material and Methods

### 2.1 Drugs

Doxycycline (#631311, Clontech, Mountain View, CA, USA) and oxaliplatin (#O9512, Sigma Chemical Company, St. Louis, MO, USA) were reconstituted in tissue‐culture‐grade water. Both drugs were filtered through a 0.22‐μm pore filter before being used in experiments.

### 2.2 Cell culture

The LoVo and HCT116 colon cancer cell lines were purchased from the American Type Culture Collection (Manassas, VA, USA). The cells were cultured and maintained in Dulbecco's modified Eagle medium containing 5 mM (1 g/L) glucose (Gibco Thermo Fisher Scientific, 11885‐084) or 25 mM (5 g/L) glucose (Gibco Thermo Fisher Scientific, 11965‐092) supplemented with 10% fetal bovine serum (Gibco) and 1% penicillin–streptomycin (Gibco) in a 37°C incubator with 5% CO_2_. CRC cells were cultured under either normal glucose (5 mM) or HG (25 mM) conditions for 7 days prior to drug treatment. The 7‐day high‐glucose treatment was selected based on previous studies indicating that mitochondrial biogenesis and related metabolic adaptations induced by hyperglycemia typically require 5–7 days to become evident in various cell types [20, 21]. After 7 days of glucose conditioning, doxycycline was added for 24 h at the indicated concentrations.

The oxaliplatin‐resistant LoVo (LoVo_OxR) cell line was provided by Dr. Shang‐Hung Chen and Prof. Hsiao‐Sheng Liu. The oxaliplatin concentration was gradually increased from 1 to 10 μM. The oxaliplatin‐resistant HCT116 (HCT116_OxR) cell line was established using the method reported by Dr. Chen [22]. For resistance induction, parental cells were chronically treated with stepwise increases in oxaliplatin concentrations, starting from 0.1 μM and gradually increasing up to 10 μM. At each concentration level, cells were maintained and passaged five times to ensure adaptation before escalation to the next concentration. The entire induction process spanned approximately 5–6 months. Both LoVo_OxR and HCT116_OxR cells were subsequently maintained in culture medium containing 10 μM oxaliplatin for long‐term culture and experiments.

### 2.3 Mitochondrial Mass Detection

Cells were trypsinised, resuspended and incubated in medium containing 100 nM dye (MitoTracker Green FM) for 30 min at 37°C. Mitochondrial mass was measured by staining the cells and analysing them through flow cytometry (Ex/Em = 488/530 nm, FC 500 MCL, Beckman Coulter Inc.). Doxycycline was added in the HG + Dox group with the concentration of 1 ng/mL in culture medium.

### 2.4 Glucose Metabolism Assay

The oxygen consumption rate (OCR) was examined using an XF HS Mini extracellular flux analyser (Agilent Technologies, Santa Clara, CA, USA). Briefly, 1 × 10^4^ cells were seeded into eight‐well Seahorse plates and incubated overnight at 37°C. For OCR (pmol/min) detection, approximately 1 μM oligomycin, 2 μM carbonyl cyanide‐p‐trifluoromethoxyphenylhydrazone and 0.5 μM rotenone were added. The OCR was normalised on the basis of the protein concentration and plotted using Wave software (Agilent Technologies).

### 2.5 Wound Healing Assay

A cell suspension was adjusted to 5 × 10^5^ cells/mL, and 80 μL was added to each well of the culture inserts (two‐well format). Once the cells reached confluence and established a monolayer, the culture inserts were removed. Following a wash with phosphate‐buffered saline, the cells were cultured in media containing 5 mM glucose (NG), 25 mM glucose (HG) or 25 mM glucose combined with 1 ng/mL doxycycline (HG + Dox). The migrated cells were imaged using a Leica DMI6000 B microscope (Leica Microsystems, Wetzlar, Germany) at 24 and 48 h, respectively. The wound closure was analysed with Image J software (National Institutes of Health).

### 2.6 Cell Counting Kit‐8 (CCK‐8) Assay

The LoVo (7.5 × 10^3^ cells/well) and HCT116 (7 × 10^3^ cells/well) cells were seeded in 96‐well plates. Following 24 h of adhesion, cells were treated with increasing concentrations of oxaliplatin (0, 0.2, 2, 20 and 200 μM) for 24 h. The treatment medium for the HG and HG + Dox groups contained HG at a concentration of 25 mM. The HG + Dox and OxR + Dox groups received combination therapy with doxycycline at a concentration of 1 pg/mL. A cell counting kit‐8 (CCK‐8, Sigma‐Aldrich, St Louis, MO, USA) was performed to assess cell viability. After treatment, 10 μL of CCK‐8 solution was added to each well. The cells were then incubated for 4 h at 37°C. The optical density (OD) was measured at 450 nm by using a microplate reader (BioTek Instrument, Winooski, VT, USA). The IC_50_ value for oxaliplatin was calculated on the basis of cell viability.

### 2.7 Western Blotting

Cells were harvested after 7 days of treatment with 5 mM glucose or 25 mM glucose, and an additional 1 ng/mL doxycycline was added on the 7th day. The cells were lysed in RIPA lysis buffer (Merck Ltd., USA, 20‐188) that contained protease inhibitors (Sigma‐Aldrich, P8215) and a phosphatase inhibitor cocktail (Sigma‐Aldrich, P8340). Protein concentrations were quantified using Protein Assay Dye (Bio‐Rad Laboratories, Hercules, CA, USA, #5000006). Protein samples (20 μg) were separated by SDS‐PAGE and transferred to a PVDF membrane (Merck Millipore, Burlington, MA, USA, IPVH00010). After blocking was conducted, the membrane was incubated with the following primary antibodies at 4°C overnight: c‐Myc (Cell Signalling Technology Inc., #18583, 1:1000), p‐PGC‐1alpha (R&D Systems, #AF6650, 1:1000), COX4 (Cell Signalling Technology Inc., #4844, 1:1000) and GAPDH (Abcam Cambridge, UK, #ab9845, 1:1000). After being washed with TBST, the membranes were incubated with secondary antibodies (Jackson ImmunoResearch Laboratories Inc.; Peroxidase AffiniPure Goat Anti‐Rabbit IgG [H+L]; RRID: AB_2313567; Catalogue number: 111‐035‐003, 1:100,000) for 1 h at room temperature. These membranes were washed five times with TBST, and protein bands were visualised using ECL substrates (Thermo Fisher Scientific, Waltham, MA, USA, #32106) with the ChemiDoc MP Imaging System (Bio‐Rad Laboratories Inc.).

### 2.8 In Vivo Xenograft Study

Seven‐week‐old male BALB/c nude mice were purchased from BioLasco Taiwan Co. Ltd. (Taipei, Taiwan). After 1 week of acclimation, hyperglycaemia was induced via a single intraperitoneal injection of streptozotocin (200 mg/kg, Sigma‐Aldrich, St. Louis, MO, USA). One week after STZ administration—upon confirmation of hyperglycaemia—mice were subcutaneously injected with LoVo‐Parental cells on the left flank and LoVo‐OxR cells on the right flank (2 × 10^6^ cells/100 μL/mouse). One week after tumour implantation, mice were randomly assigned to three treatment groups: (I) vehicle, (II) oxaliplatin and (III) oxaliplatin + doxycycline. Treatments were administered for four consecutive weeks. Mice were housed in a specific pathogen‐free environment throughout the study. Blood glucose levels were monitored once weekly throughout the 6‐week experimental period. Approximately 10 μL of blood was collected via tail vein puncture, and glucose levels were measured using the ACCU‐CHEK glucometer and test strips (ROCHE Diagnostics). The treatment was started 7 days after completion of the injections. The doses and schedules for oxaliplatin and doxycycline were based on those in previous studies [23, 24]. Oxaliplatin (5 mg/kg) and doxycycline (50 mg/kg) were administered every 3 days for 4 weeks. Tumour diameters were measured three times per week, and tumour volume (mm^3^) was calculated using the following formula: volume = [length × width^2^]/2. The mice were sacrificed 1 week after the final chemotherapy dose under anaesthesia with isoflurane followed by cardiac puncture. The in vivo study followed the protocols approved by the Institutional Animal Care and Use Committee of Kaohsiung Medical University in accordance with the guidelines for the Care and Use of Laboratory Animals.

### 2.9 Tumour Tissues

This study included 21 de‐link tumour tissues with primary CRC in UICC stages III, classified into two groups on the basis of postoperative relapse after adjuvant oxaliplatin‐based chemotherapy. The first group consisted of 11 de‐link tumour tissues with CRC and hyperglycaemia who experienced relapse; whereas the second group comprised 10 de‐link tumour tissues with CRC and normoglycemia who did not experience relapse. Blood sugar level was defined in accordance with the 2003 American Diabetes Association diagnostic criteria for diabetes (normoglycemia: < 110 mg/dL; hyperglycaemia: ≥ 110 mg/dL) [25]. The retrospective study protocol was approved by the Institutional Review Board of Kaohsiung Medical University Hospital (KMUHIRB‐G(I)‐20190042).

### 2.10 Statistical Analysis

All experiments were performed in triplicate. Data are presented as the mean ± standard error of the mean. Statistical data were analysed using one‐way analysis of variance with SPSS 20‐Windows (IBM Corp., USA). A *p* value of < 0.05 was considered statistically significant.

## 3 Results

### 3.1 HG Promoted Mitochondrial Biogenesis, OCR and Cell Migration; Doxycycline Reversed Mitochondrial Biogenesis Induced by HG

**FIGURE 1:**
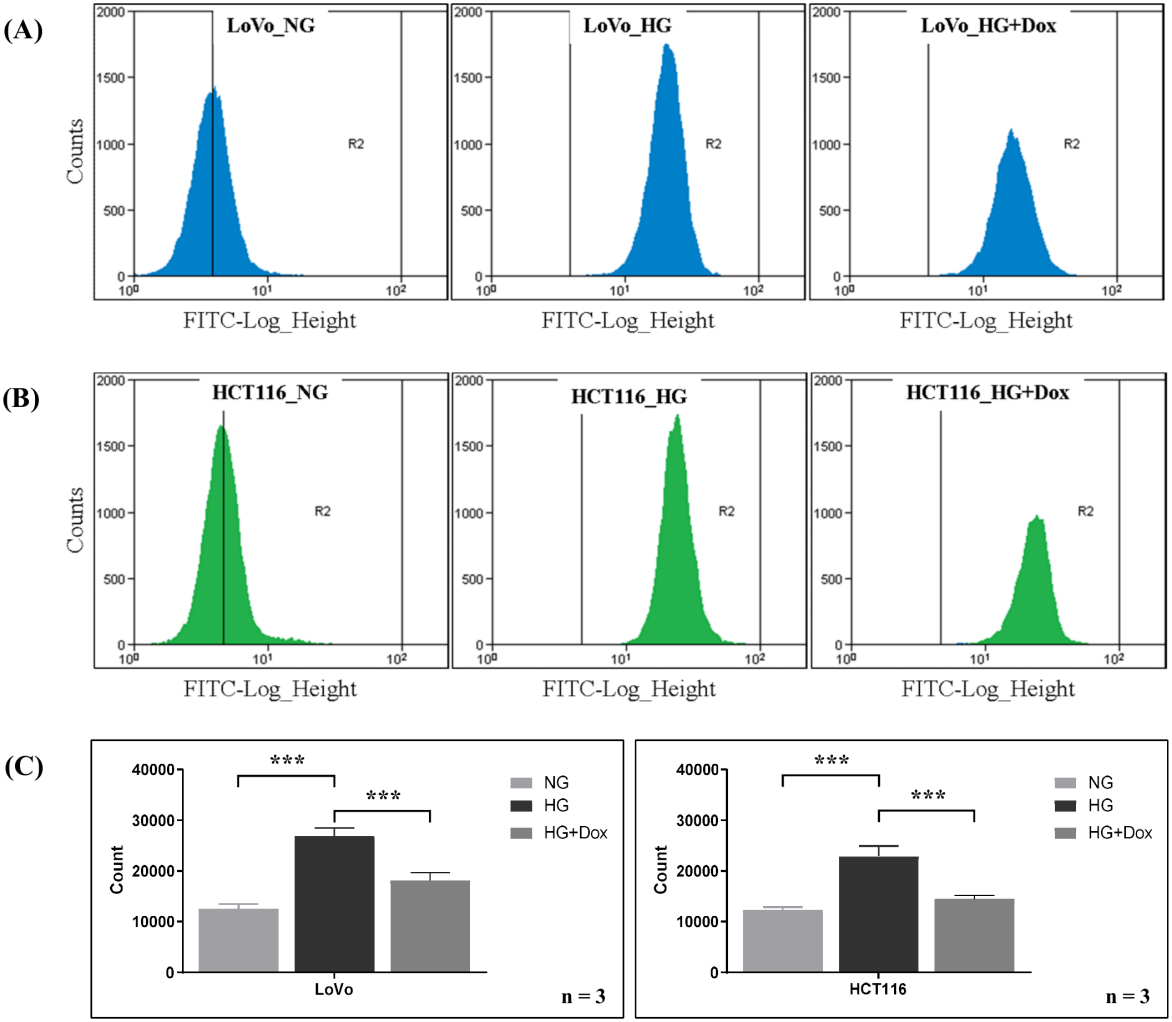
HG promotes mitochondrial biogenesis in LoVo and HCT116 cells, whereas doxycycline reduces mitochondrial mass. Mitochondrial mass was measured using flow cytometry in (A) LoVo and (B) HCT116 cells. (C) The mitochondrial mass was higher in the HG group than in the normal glucose group. The HG + Dox group demonstrated that doxycycline reduced mitochondrial mass. Error bars represent the mean ± SD. Data were analysed using one‐way ANOVA. ***p < 0.001.

To investigate the effects of HG and doxycycline on mitochondria in CRC cells, we treated LoVo and HCT116 cells with normal glucose (5 mM) and HG (25 mM) concentrations for 7 days. Mitochondrial biogenesis was assessed by measuring mitochondrial mass in cells through flow cytometry. In both LoVo and HCT116 cells, mitochondrial mass increased in the HG group and decreased in the group treated with doxycycline (Figure 1, *p* < 0.01). The results indicate that HG enhances mitochondrial biogenesis and that doxycycline reverses this effect.

**FIGURE 2:**
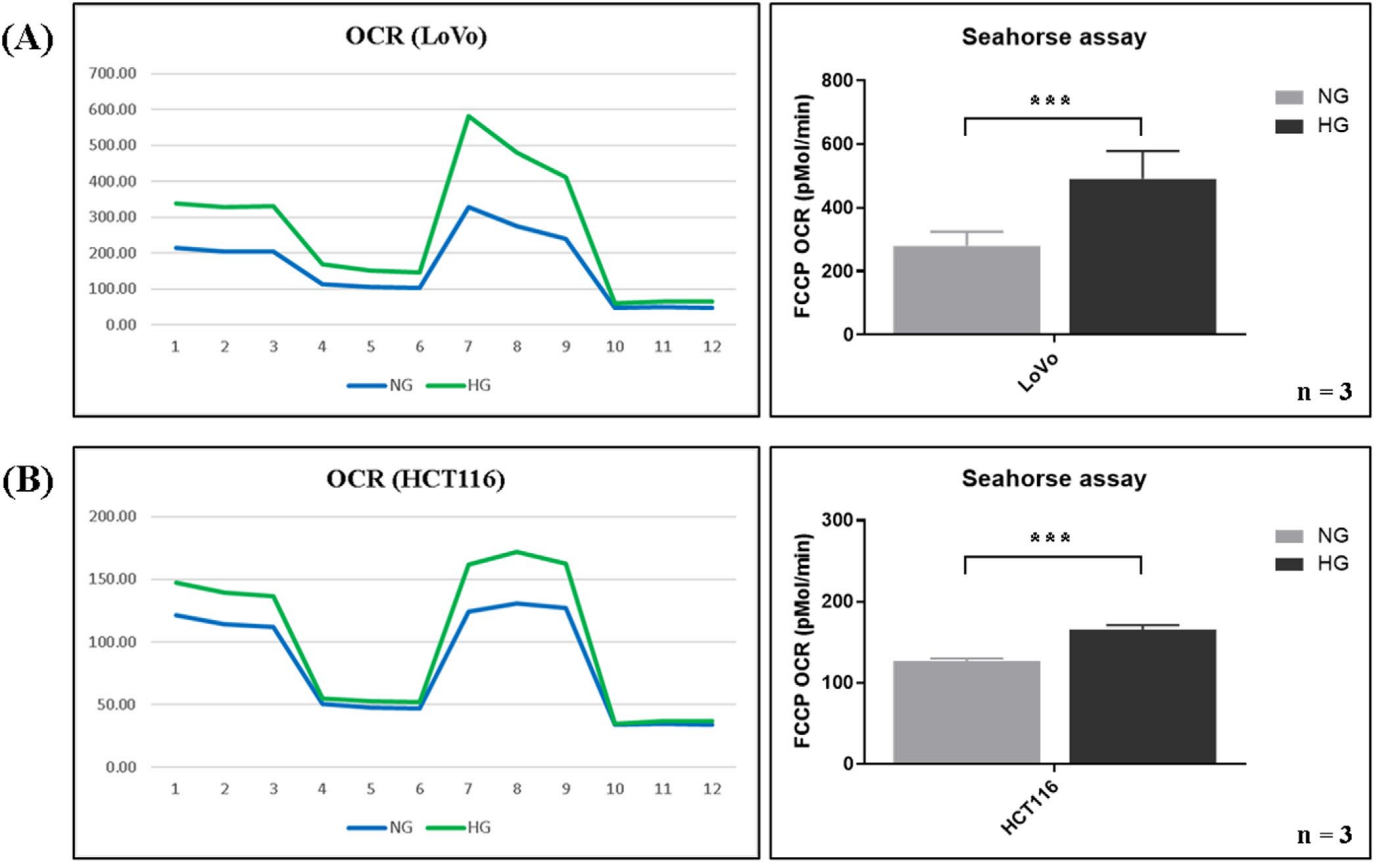
HG increased the OCR in (A) LoVo and (B) HCT116 cells. To evaluate changes in the aerobic respiratory potential of CRC cells under HG and normal glucose (NG) conditions, the OCR was analysed using a Seahorse XF analyser. The HG group had a higher OCR than did the NG group in both LoVo and HCT116 cells. Error bars represent the mean ± SD. Data were analysed using Student's *t* test. ***p < 0.001.

Next, we examined whether HG increased respiratory capacity in colon cancer cells by measuring the OCR. The OCR was significantly increased in the HG groups for both LoVo and HCT116 cells (Figure 2, *p* < 0.01).

**FIGURE 3:**
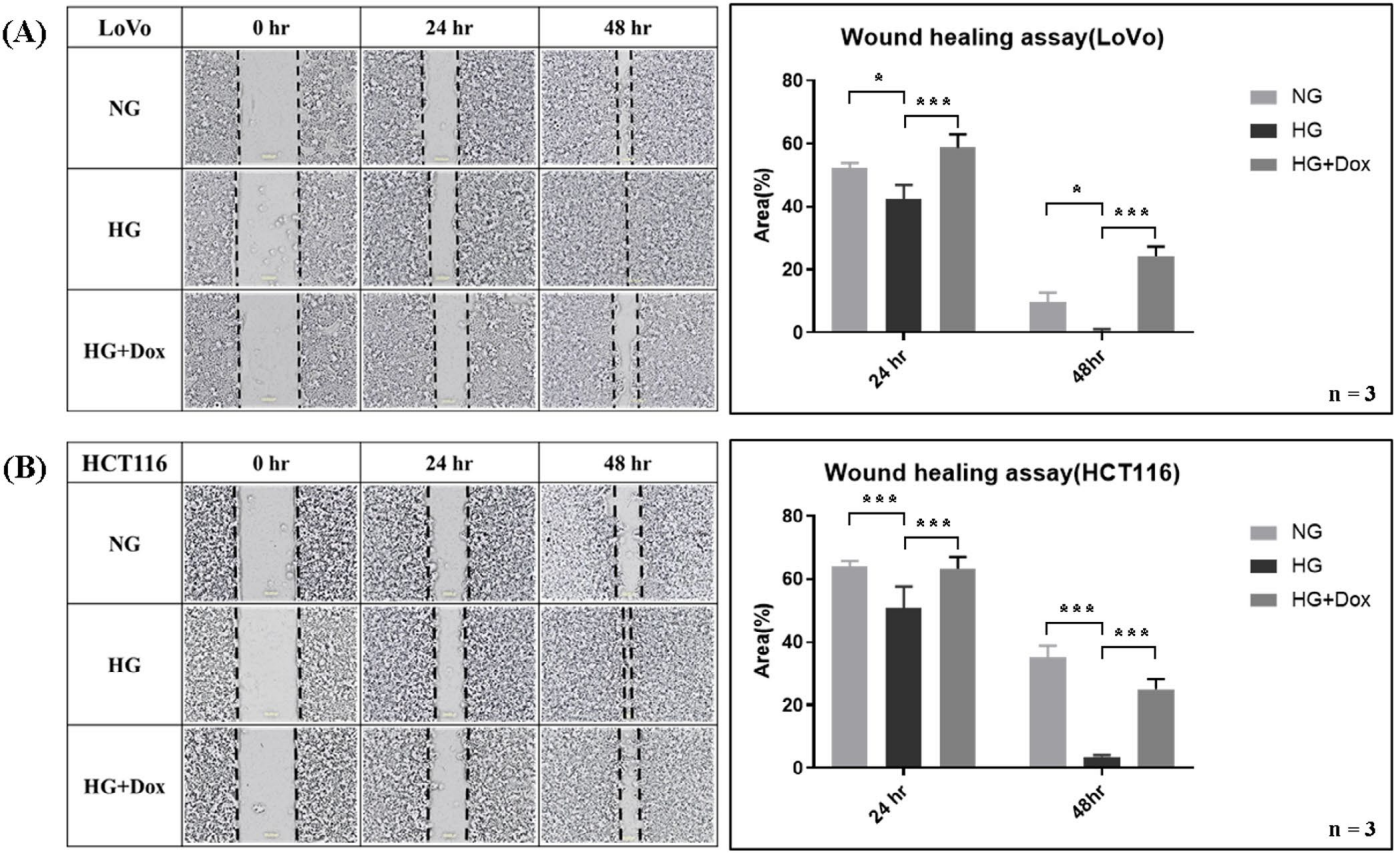
HG promoted cell migration, whereas doxycycline reversed the effect of HG in (A) LoVo and (B) HCT116 cells. To determine cell migration under the HG condition, the wound healing assay was performed using LoVo and HCT116 cells. HG increased migration capability, as indicated by a narrower gap at 24 and 48 h. By contrast, addition of doxycycline to the HG group reduced migration, as indicated by a wider gap at 24 and 48 h. Error bars represent the mean ± SD. Data were analysed using one‐way ANOVA. *p < 0.05, ***p < 0.001.

The effects of HG and doxycycline on the migration ability of CRC cells were examined using a wound healing assay. After 24–48 h of treatment, HG had markedly increased the migration rate, whereas doxycycline had reversed this effect in both LoVo and HCT116 cells (Figure 3, both *p* < 0.05). In conclusion, HG significantly increased the OCR and cell migration by enhancing mitochondrial biogenesis, whereas doxycycline reduced the mitochondrial biogenesis induced by HG.

### 3.2 Both HG and Acquired Oxaliplatin Resistance Enhanced Oxaliplatin Resistance, and Doxycycline Counteracted This Effect

**FIGURE 4:**
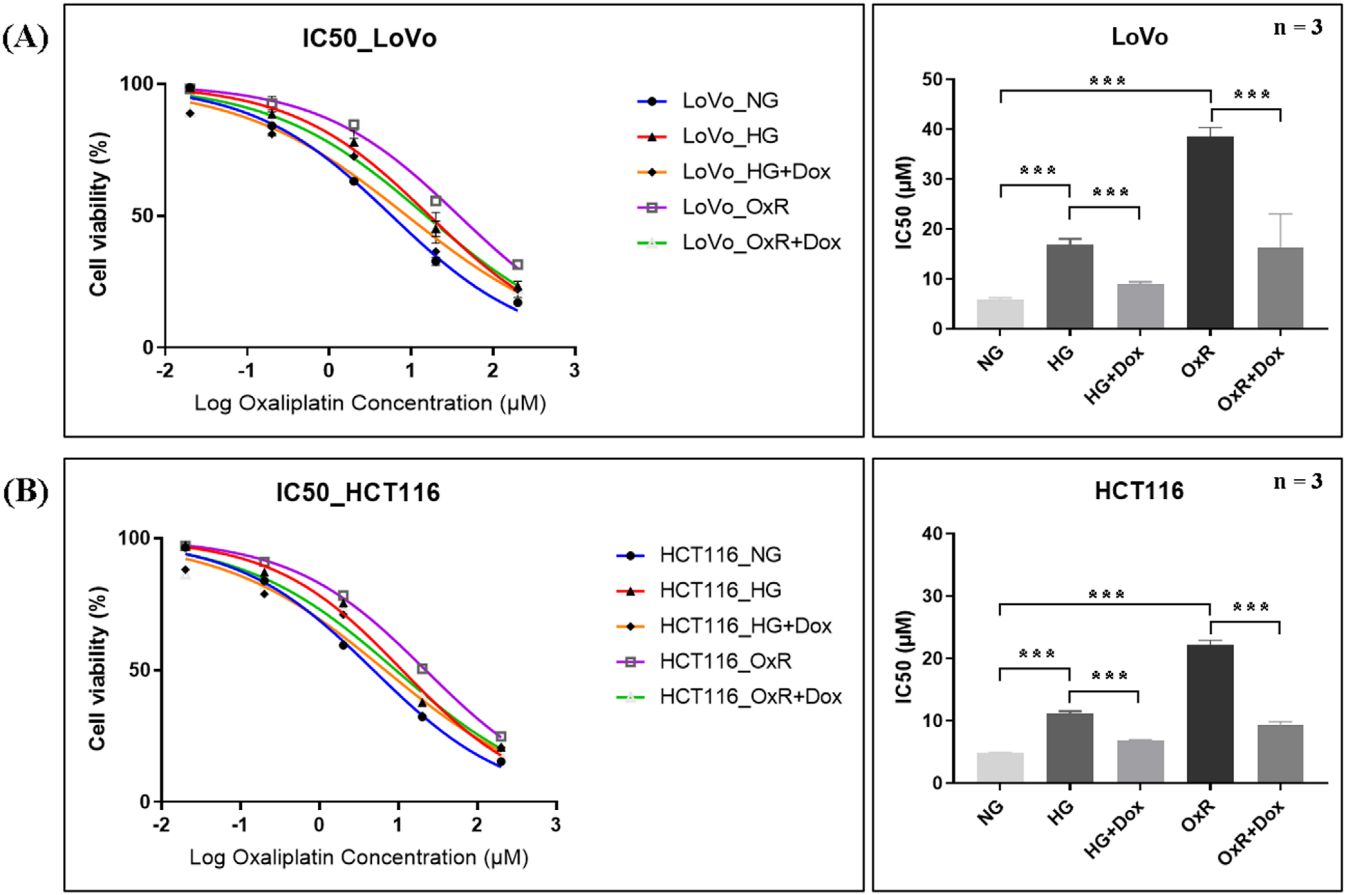
Doxycycline reverses oxaliplatin resistance induced by high glucose (HG) and acquired resistance (OxR) in CRC cells. IC_50_ values of oxaliplatin were measured using the CCK‐8 assay in LoVo and HCT116 cells under five conditions: normal glucose (NG), high glucose (HG), HG with doxycycline (HG + Dox), oxaliplatin‐resistant (OxR), and OxR with doxycycline (OxR + Dox). In both cell lines, the HG and OxR groups showed significantly elevated IC_50_ values, confirming increased resistance. Treatment with doxycycline markedly reduced the IC_50_ in both models. LoVo cells (IC_50_, μM): NG: 5.83 ± 0.40; HG: 16.85 ± 1.22; HG + Dox: 8.95 ± 0.45; OxR: 38.55 ± 1.85; OxR + Dox: 16.36 ± 6.67. HCT116 cells (IC_50_, μM): NG: 4.83 ± 0.06; HG: 11.15 ± 0.39; HG + Dox: 6.80 ± 0.06; OxR: 22.12 ± 0.81; OxR + Dox: 9.29 ± 0.58. Data are presented as mean ± SD. Statistical analysis was performed using one‐way ANOVA. ***p < 0.001.

To evaluate changes in oxaliplatin resistance in LoVo and HCT116 cells under HG and acquired oxaliplatin‐resistance conditions, we assessed cell viability by using the CCK‐8 assay to calculate the IC_50_, representing oxaliplatin resistance. Both HG and OxR cells exhibited elevated IC_50_ values compared to their normal glucose (NG) parental counterparts, confirming increased oxaliplatin resistance. However, in the groups treated with doxycycline, oxaliplatin resistance was significantly decreased in both LoVo and HCT116 cells (Figure 4, both *p* < 0.001). Doxycycline thus effectively reversed the oxaliplatin resistance induced by either hyperglycemic conditions or chronic drug exposure in CRC cells.

### 3.3 HG and Acquired Oxaliplatin Resistance Upregulated the Expression of p‐PGC‐1α and COX4 Proteins, and Doxycycline Reversed Their Overexpression

**FIGURE 5:**
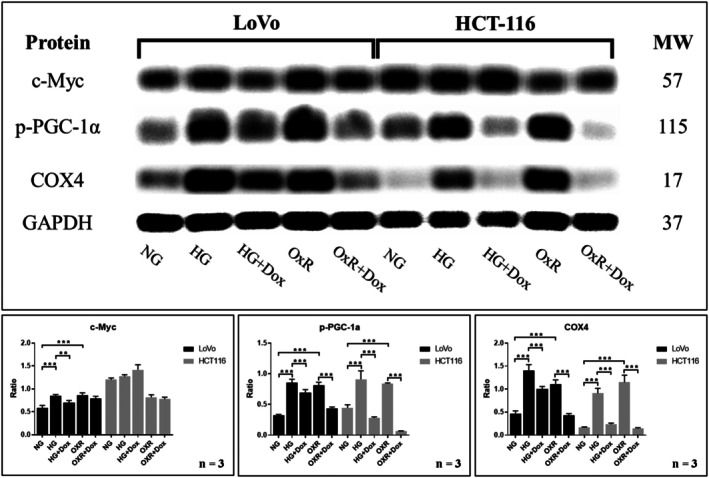
HG and OxR upregulated the expression of p‐PGC‐1α and COX4 proteins in LoVo and HCT116 cells, whereas doxycycline downregulated p‐PGC‐1α and COX4 expression induced by HG and OxR. To measure the expression of c‐Myc, p‐PGC‐1α and COX4 proteins, Western blot analysis was performed using LoVo and HCT116 cells. In both cells, the expression of p‐PGC‐1α, and COX4 proteins was upregulated in the HG group and downregulated in the HG + doxycycline (Dox) group (*p* < 0.05), also the expression of p‐PGC‐1α and COX4 proteins was upregulated in the OxR group and downregulated in the OxR + Dox group (*p* < 0.05). Error bars represent the mean ± SD. Data were analysed using the one‐way ANOVA. **p < 0.01, ***p < 0.001.

To confirm that the increased oxaliplatin resistance was associated with enhanced mitochondrial biogenesis, we evaluated the levels of c‐Myc, p‐PGC‐1α and COX4 expression in CRC cells under HG conditions and in oxaliplatin‐acquired resistance (OxR) cells by using Western blot analysis. The expression of p‐PGC‐1α and COX4 was significantly increased in both the HG and OxR groups but decreased in the HG + Dox and OxR + Dox groups for LoVo and HCT116 cells, respectively (all *p* < 0.05 or *p* < 0.001, Figure 5). The results indicated that mitochondrial biogenesis was increased given oxaliplatin resistance and decreased when doxycycline was administered in CRC cells.

### 3.4 Tumour Growth Was Suppressed by the Combination of Doxycycline and Oxaliplatin in Hyperglycaemic BALB/c Nude Mice

**FIGURE 6:**
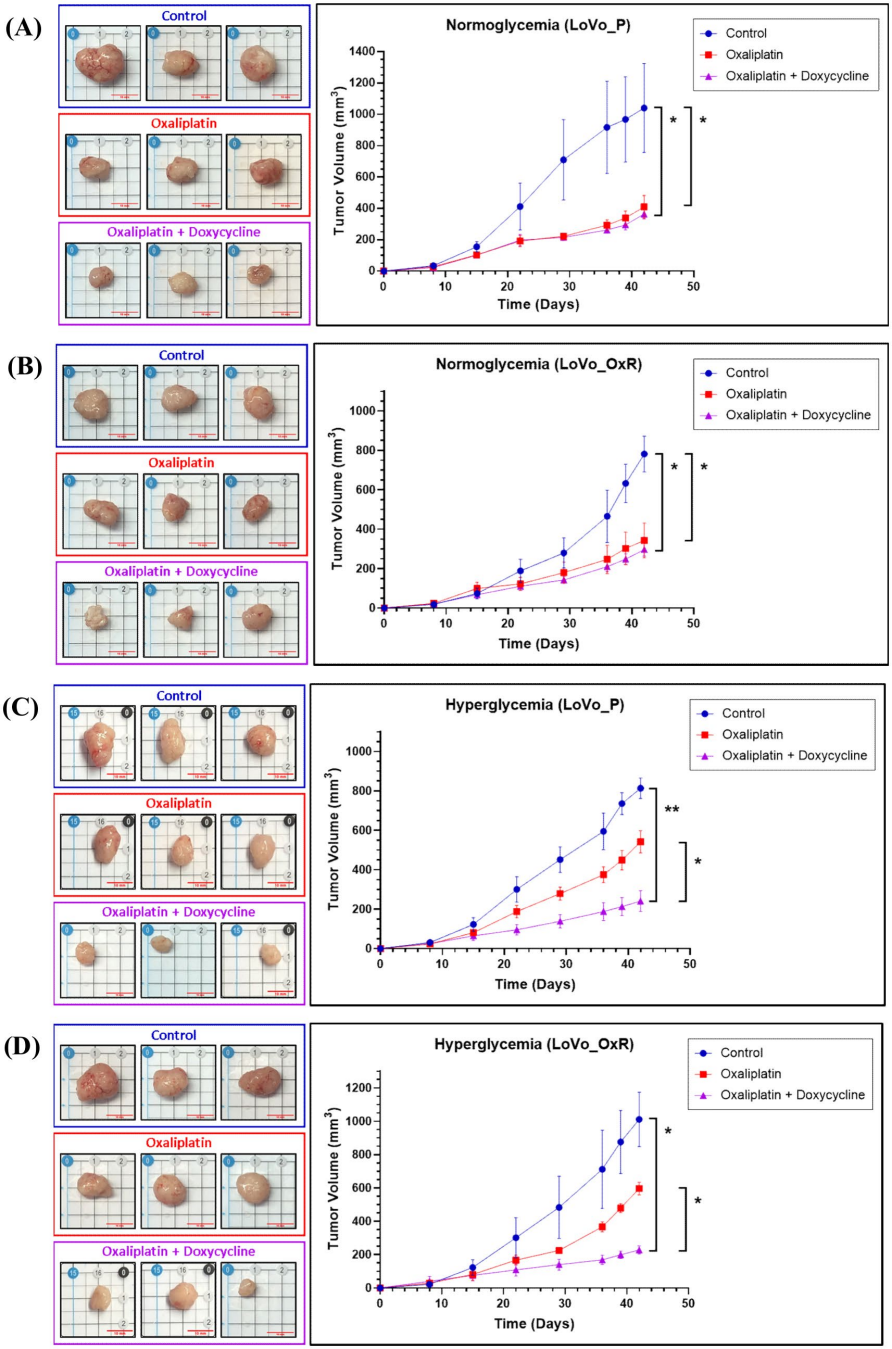
Combination of oxaliplatin and doxycycline reduced tumour volume of OxR cells in hyperglycaemic nude mice but not in normoglycaemic nude mice. LoVo_P and LoVo_OxR cells were injected subcutaneously into the left and right flank of normoglycaemic and hyperglycaemic BALB/c nude mice. Tumour volumes of LoVo_P cells (A) and LoVo_OxR cells (B) were significantly reduced by oxaliplatin alone and the combination of doxycycline and oxaliplatin in normoglycaemic nude mice (all *p* < 0.05). However, no difference was found between oxaliplatin alone and the combination treatment. Tumour volumes of LoVo_P cells (C) and LoVo_OxR cells (D) were significantly decreased following the combined doxycycline and oxaliplatin treatment in hyperglycaemic nude mice (both *p* < 0.05) but not after oxaliplatin treatment alone. A significant difference was found between oxaliplatin alone and the combined doxycycline and oxaliplatin treatment. Error bars represent the mean ± SD. Data were analysed using repeated measures ANOVA. *p < 0.05, **p < 0.01.

To assess whether doxycycline could reverse the oxaliplatin resistance enhanced by hyperglycaemia in vivo, parental LoVo cells (LoVo_P) and oxaliplatin‐resistant LoVo cells (LoVo_OxR) were subcutaneously injected into the left and right flanks of normoglycemic and hyperglycaemic BALB/c nude mice. The hyperglycaemic group exhibited significantly elevated blood glucose concentrations compared to the normoglycemic group, with mean values of 142.8 ± 39.6 mg/dL and 102.0 ± 12.8 mg/dL, respectively (data not shown). In normoglycemic mice (Figure 6A,B), tumour volumes of both LoVo_P and LoVo_OxR cells were significantly reduced by oxaliplatin alone as well as by the combination of doxycycline and oxaliplatin (all *p* < 0.05). However, no significant difference was discovered between oxaliplatin alone and the combination treatment (*p* > 0.05). In hyperglycaemic mice (Figure 6C,D), the combination of doxycycline and oxaliplatin significantly reduced the tumour volumes of both LoVo_P and LoVo_OxR cells (both *p* < 0.05), whereas oxaliplatin alone had no significant effect on either cell line (both *p* > 0.05). A notable difference in tumour volume reduction was noted between oxaliplatin alone and the combination treatment (both *p* < 0.05, Figure 6C,D). These results indicated that the combined treatment of doxycycline and oxaliplatin did not enhance therapeutic efficacy in normoglycemic mice but markedly inhibited tumour growth in hyperglycaemic mice.

### 3.5 C‐Myc, p‐PGC‐1α and COX4 Were Significantly Overexpressed in Tumour Tissues With Hyperglycaemia and CRC Receiving Adjuvant Chemotherapy Who Experienced Relapse

**FIGURE 7:**
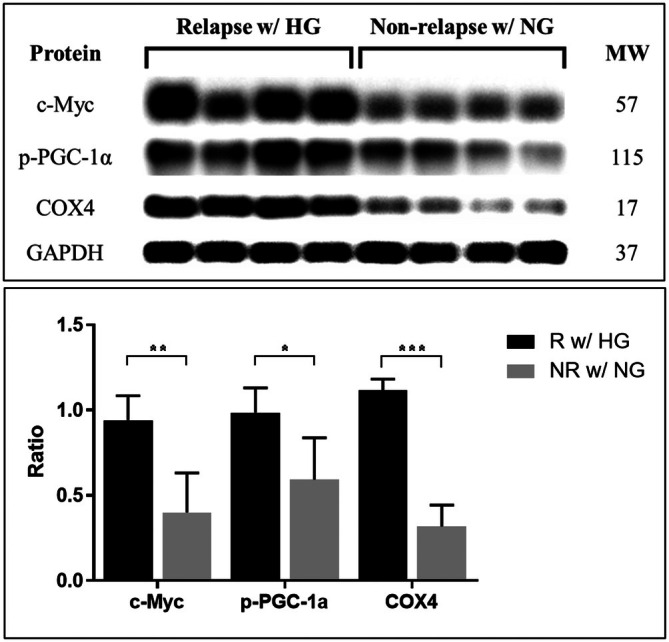
c‐Myc, p‐PGC‐1α and COX4 proteins were significantly highly expressed in tumour tissues with CRC and hyperglycaemia who experienced relapse. To measure the expression of c‐Myc, p‐PGC1α and COX4 protein, Western blot analysis was performed using tissues from tumour tissues with stage III CRC. The expression of c‐Myc, p‐PGC‐1α and COX4 proteins was significantly higher in tumour tissues with CRC and hyperglycaemia who experienced relapse than in tumour tissues with CRC and normoglycaemia who did not experience relapse (*p* = 0.007, 0.033 and < 0.001, respectively). Error bars represent the mean ± SD. Data were analysed using one‐way ANOVA. *p < 0.05, **p < 0.01, ***p < 0.001.

To extend the in vivo findings, we determined the levels of c‐Myc, p‐PGC‐1α and COX4 in stage III CRC tumour tissues through Western blotting. The analysis included samples from 11 tumour tissues with hyperglycaemia and CRC who experienced relapse and 10 tumour tissues with CRC and normoglycaemia who did not experience relapse. C‐Myc, p‐PGC‐1α and COX4 were significantly more highly expressed in tumour tissues with hyperglycaemia and CRC who experienced relapse than in tumour tissues with CRC and normoglycaemia who did not experience relapse (*p* = 0.007, 0.033 and < 0.001, Figure 7). These findings indicated that c‐Myc, p‐PGC‐1α and COX4 were all upregulated in tumour tissues with CRC with chemoresistance undergoing adjuvant chemotherapy.

## 4 Discussion

In Taiwan, the development of resistance to adjuvant chemotherapy remains a major obstacle in the treatment of CRC. Chemoresistance often leads to low therapeutic efficacy and poor patient outcomes [26]. One potential mechanism underlying this chemoresistance is increased mitochondrial biogenesis in cancer cells, which enhances their survival in the face of chemotherapeutic agents [5]. This phenomenon limits the effectiveness of conventional treatments, such as oxaliplatin, in patients with CRC [27]. Understanding the molecular pathways that contribute to chemoresistance is crucial for developing more effective therapeutic strategies to overcome these clinical challenges.

HG affects the efficacy of chemotherapy [28]. Hyperglycaemia promotes cancer cell proliferation and enhances resistance to chemotherapeutic agents, such as oxaliplatin, by upregulating mitochondrial biogenesis and oxidative capacity [29, 30]. In addition, hyperglycaemia is associated with poor clinical outcomes in patients with cancer, potentially due to its role in promoting tumour progression and reducing the effectiveness of chemotherapy [31].

Tetracyclines have long been known for their antimicrobial properties, with doxycycline being notable for its long duration of action and various anticancer properties, particularly cytotoxic and antiproliferative effects. Doxycycline, a tetracycline antibiotic, has garnered attention for its ability to inhibit mitochondrial biogenesis by downregulating PGC‐1α, a key regulator of mitochondrial function. This disruption of mitochondrial activity, which is crucial for cancer cell survival under metabolic stress such as chemotherapy, weakens cancer cells [32]. Recent studies have demonstrated that doxycycline reduces both the proliferation of mitochondria and the oxidative capacity of cancer cells [33]. Decreased mitochondrial function results in cancer cells being more vulnerable to chemotherapeutic agents, making them less capable of surviving the oxidative stress and DNA damage caused by oxaliplatin [34]. Sagar et al. reported that doxycycline reduces cellular proliferation, increases cytotoxicity, and may induce apoptosis through a mitochondria‐mediated pathway [35].

In the present study, we identified the chemoresistance effect of HG and the reversal effect of doxycycline in CRC cell lines and an animal model. Our in vitro studies demonstrated that HG enhanced mitochondrial biogenesis, oxygen consumption, migration and oxaliplatin resistance and that doxycycline reversed these effects. The potential molecular mechanism underlying HG‐induced mitochondrial biogenesis involved the upregulation of p‐PGC‐1α and COX4 expression, which was reversed by doxycycline administration. Although our study did not include Co‐IP or ChIP assays to directly validate the functional interaction between c‐Myc and p‐PGC‐1α, previous studies have reported mechanistic links between these two molecules. For example, Li et al. and Bhalla et al. demonstrated that c‐Myc interacts with PGC‐1 coactivators to regulate mitochondrial gene expression and promote oxidative metabolism in cancer cells [36, 37]. A comprehensive review by Stine et al. further emphasised the role of c‐Myc in orchestrating mitochondrial biogenesis and metabolic reprogramming via transcriptional regulation of PGC‐1α‐related pathways [38]. These findings support our hypothesis that c‐Myc may regulate p‐PGC‐1α activity in the context of hyperglycaemia‐induced chemoresistance. Future studies incorporating Co‐IP or ChIP analyses will help to further confirm this regulatory mechanism.

We hypothesise that, in animal models, doxycycline reverses the enhanced mitochondrial biogenesis and oxaliplatin resistance under HG conditions. In the present study, we found that oxaliplatin exhibited therapeutic efficacy under normoglycemic conditions, in which mitochondrial biogenesis was not increased. Thus, doxycycline administration did not provide any additional synergistic benefit under these conditions. By contrast, oxaliplatin exhibited limited therapeutic efficacy under hyperglycaemic conditions, whereas HG increased mitochondrial biogenesis and oxaliplatin resistance. However, the combination of doxycycline and oxaliplatin significantly improved therapeutic outcomes because doxycycline counteracted HG‐induced mitochondrial biogenesis, thereby reversing resistance and enhancing oxaliplatin's overall efficacy. In addition, in our in vivo studies, we confirmed that p‐PGC‐1α and COX4 protein levels were higher in tumour tissues from CRC patients with hyperglycaemia who experienced relapse than in tumour tissues from CRC patients with normoglycemia who did not experience relapse.

Overall, the findings indicate that HG induces the overexpression of p‐PGC‐1α and COX4, promoting chemoresistance in CRC cells by enhancing mitochondrial biogenesis. Repurposing doxycycline can overcome this chemoresistance. This aligns with recent research showing that tetracycline derivatives such as doxycycline not only inhibit mitochondrial protein synthesis but also enhance antitumour T‐cell immunity via the Zap70 signalling pathway, highlighting their dual role in targeting both cancer cell metabolism and immune modulation [39]. Zhu et al. summarised the synergistic antitumour effects of metformin when combined with chemotherapy, targeted therapy, radiotherapy and immunotherapy, highlighting its mitochondrial complex I inhibition and immune modulatory roles [40]. Moreover, Julius et al. highlighted the intricate interplay between hyperglycaemia, cancer cell metabolism, and the tumour microenvironment, reinforcing the rationale for targeting mitochondrial pathways and metabolic regulators in hyperglycaemic CRC patients [41]. In summary, we suggest that the combination of doxycycline and oxaliplatin be considered as an adjunctive therapy to improve outcomes in stage III CRC patients undergoing adjuvant chemotherapy. However, further clinical trials are necessary to confirm the clinical relevance of these findings.

Our study also contributes to the growing body of literature linking altered cancer metabolism—particularly mitochondrial biogenesis and oxidative phosphorylation—with drug resistance. By targeting mitochondrial pathways, doxycycline disrupts the metabolic adaptations exploited by chemoresistant cancer cells, representing a promising strategy within the field of metabolic oncology.

In parallel, the repurposing of antibiotics such as doxycycline for anticancer purposes offers a practical and cost‐effective therapeutic avenue. Nonetheless, it is important to recognise potential risks associated with the noninfectious use of antibiotics. Excessive or prolonged use could contribute to the development of antimicrobial resistance, a serious global health concern. Future clinical translation of antibiotic‐based strategies must therefore include careful consideration of dosage, treatment duration and antibiotic stewardship to ensure both efficacy and safety.

## Author Contributions

**Chang‐Han Wu:** writing – original draft (equal). **Ching‐Wen Huang:** writing – original draft (equal). **Yen‐Cheng Chen:** formal analysis (equal). **Kwan‐Ling Yip:** methodology (equal). **Zhi‐Feng Miao:** methodology (equal). **Wei‐Chih Su:** investigation (equal). **Tsung‐Kun Chang:** validation (equal). **Hsiang‐Lin Tsai:** supervision (equal). **Yung‐Sung Yeh:** formal analysis (equal). **Hsiao‐Sheng Liu:** supervision (equal). **Jaw‐Yuan Wang:** writing – review and editing (equal).

## Conflicts of Interest

The authors declare no conflicts of interest.

## Supporting information


**Appendix S1:** jcmm70795‐sup‐0001‐AppendixS1.docx.

## Data Availability

The data that support the findings of this study are available on request from the corresponding author. The data are not publicly available due to privacy or ethical restrictions.
