# Supplementary material for: Repurposing Doxycycline to Overcome High‐Glucose–Induced Mitochondrial Biogenesis–Mediated Chemoresistance in Colorectal Cancer Cells
Source: J Cell Mol Med. 2025 Oct 13;29(19):e70795. doi: 10.1111/jcmm.70795 (PMC12516229; doi:10.1111/jcmm.70795)
Supplement: Supplementary file 1 — Appendix S1: jcmm70795‐sup‐0001‐AppendixS1.docx. [file JCMM-29-e70795-s001.docx]

**Figure Legends**

**Figure 1. HG promotes mitochondrial biogenesis in LoVo and HCT116 cells, whereas doxycycline reduces mitochondrial mass.** Mitochondrial mass was measured using flow cytometry in (A) LoVo and (B) HCT116 cells. (C) The mitochondrial mass was higher in the HG group than in the normal glucose group. The HG+Dox group demonstrated that doxycycline reduced mitochondrial mass. Error bars represent the mean ± SD. Data were analysed using one-way ANOVA. **: *p* < 0.01; ***: *p* < 0.001.

**Figure 2.** **HG increased the OCR in (A) LoVo and (B) HCT116 cells.** To evaluate changes in the aerobic respiratory potential of CRC cells under HG and normal glucose (NG) conditions, the OCR was analysed using a Seahorse XF analyser. The HG group had a higher OCR than did the NG group in both LoVo and HCT116 cells. Error bars represent the mean ± SD. Data were analysed using Student’s t test. ***: *p* < 0.001.

**Figure 3. HG promoted cell migration, whereas doxycycline reversed the effect of HG in (A) LoVo and (B) HCT116 cells.** To determine cell migration under the HG condition, the wound healing assay was performed using LoVo and HCT116 cells. HG increased migration capability, as indicated by a narrower gap at 24 and 48 h. By contrast, addition of doxycycline to the HG group reduced migration, as indicated by a wider gap at 24 and 48 h. Error bars represent the mean ± SD. Data were analysed using one-way ANOVA. *: *p* < 0.05; ***: *p* < 0.001.

**Figure 4. Doxycycline reverses oxaliplatin resistance induced by high glucose (HG) and acquired resistance (OxR) in CRC cells.**

IC50 values of oxaliplatin were measured using the CCK-8 assay in LoVo and HCT116 cells under five conditions: normal glucose (NG), high glucose (HG), HG with doxycycline (HG+Dox), oxaliplatin-resistant (OxR), and OxR with doxycycline (OxR+Dox). In both cell lines, the HG and OxR groups showed significantly elevated IC50 values, confirming increased resistance. Treatment with doxycycline markedly reduced the IC50 in both models.

**LoVo cells (IC50, μM):** NG: 5.83 ± 0.40; HG: 16.85 ± 1.22; HG+Dox: 8.95 ± 0.45; OxR: 38.55 ± 1.85; OxR+Dox: 16.36 ± 6.67

**HCT116 cells (IC50, μM):** NG: 4.83 ± 0.06; HG: 11.15 ± 0.39; HG+Dox: 6.80 ± 0.06; OxR: 22.12 ± 0.81; OxR+Dox: 9.29 ± 0.58

Data are presented as mean ± SD. Statistical analysis was performed using one-way ANOVA. ***p < 0.001.

**Figure 5. HG and OxR upregulated the expression of p-PGC-1α and COX4 proteins in LoVo and HCT116 cells, whereas doxycycline downregulated p-PGC-1α and COX4 expression induced by HG and OxR.** To measure the expression of c-Myc, p-PGC-1α, and COX4 proteins, Western blot analysis was performed using LoVo and HCT116 cells. In both cells, the expression of p-PGC-1α, and COX4 proteins was upregulated in the HG group and downregulated in the HG+doxycycline (Dox) group (*P* < 0.05), also the expression of p-PGC-1α and COX4 proteins was upregulated in the OxR group and downregulated in the OxR+Dox group (*P* < 0.05). Error bars represent the mean ± SD. Data were analysed using the one-way ANOVA. *: *p* < 0.05, **: *p* < 0.01, ***: *p* < 0.001.

**Figure 6. Combination of oxaliplatin and doxycycline reduced tumour volume of OxR cells in hyperglycaemic nude mice but not in normoglycaemic nude mice.** LoVo_P and LoVo_OxR cells were injected subcutaneously into the left and right flank of normoglycaemic and hyperglycaemic BALB/c nude mice. Tumour volumes of LoVo_P cells (A) and LoVo_OxR cells (B) were significantly reduced by oxaliplatin alone and the combination of doxycycline and oxaliplatin in normoglycaemic nude mice (all *P* < 0.05). However, no difference was found between oxaliplatin alone and the combination treatment. Tumour volumes of LoVo_P cells (C) and LoVo_OxR cells (D) were significantly decreased following the combined doxycycline and oxaliplatin treatment in hyperglycaemic nude mice (both *p* < 0.05) but not after oxaliplatin treatment alone. A significant difference was found between oxaliplatin alone and the combined doxycycline and oxaliplatin treatment. Error bars represent the mean ± SD. Data were analysed using repeated measures ANOVA. *: *p* < 0.05, **: *p* < 0.01.

**Figure 7. c-Myc, p-PGC-1α and COX4 proteins were significantly highly expressed in tumor tissues with CRC and hyperglycaemia who experienced relapse.** To measure the expression of c-Myc, p-PGC1α, and COX4 protein, Western blot analysis was performed using tissues from tumor tissues with stage III CRC. The expression of c-Myc, p-PGC-1α, and COX4 proteins was significantly higher in tumor tissues with CRC and hyperglycaemia who experienced relapse than in tumor tissues with CRC and normoglycaemia who did not experience relapse (*p* = 0.007, 0.033, and <0.001, respectively). Error bars represent the mean ± SD. Data were analysed using one-way ANOVA. *: *p* < 0.05; **: *p* < 0.01; ***: *p* < 0.001.
